# Advances in the application of human-machine collaboration in healthcare: insights from China

**DOI:** 10.3389/fpubh.2025.1507142

**Published:** 2025-02-05

**Authors:** Wuzhen Wang, Liangji Liu

**Affiliations:** School of Clinical Medicine, Jiangxi University of Chinese Medicine, Nanchang, Jiangxi, China

**Keywords:** human-machine collaboration and interaction, artificial intelligence in healthcare, chronic disease management, health management, medical education, precision healthcare and traditional Chinese medicine

## Abstract

In the context of the technological revolution and the digital intelligence era, the contradiction between the rising incidence of diseases and the uneven distribution of quality medical resources is highlighted, and the diagnosis and prevention of diseases, and the prognosis and management of diseases are particularly important in the current society of aging population. “Human–machine collaboration” is based on an intelligent algorithmic system that utilizes the complementary strengths of humans and machines for data exchange, task allocation, decision making and collaborative work to provide more decision support. The traditional healthcare model is highly dependent on the unified management of hospitals, which further increases the burden on the healthcare system and often makes it difficult to formulate and implement personalized and precise rehabilitation programs for patients, which seriously affects their prognosis and quality of life, and increases the risk of re-admission to hospitals. In view of this, human-computer collaboration, an innovation-driven technology, is a groundbreaking solution to the outstanding healthcare issues of today. We use the subject words “Human–machine collaboration” OR “Human-Computer Interaction” OR “HCI” AND “chronic disease” OR “Health management” OR ”Precision medicine “were searched for CNKI, Wanfang Data, VIP, CBM, PubMed, Web of science, Embase, Cochrane Library and other Chinese and English databases to identify all relevant studies and compare their results, and finally include 68 relevant literature articles, we identified the broad application of HCI in five main areas: disease screening and treatment, health management, medical education, traditional medicine, and the integration and processing of medical data. The aim is to review the concept of human-computer collaboration, its application in global healthcare environments, and the challenges it faces, with a view to continually driving innovation in healthcare models, optimizing the allocation of healthcare resources, and providing new paradigms for the development and application of innovative technologies in healthcare.

## 1 Introduction

Pandemics such as COVID-19, Ebola disease, AIDS and others put unprecedented pressure on global health systems (1), requiring new solutions and strategies to address challenges and seize opportunities, while also accelerating the development of telemedicine and digital health solutions. In 2000, the World Health Organization (WHO) established the Global Outbreak Alert and Response Network (2). The goal of GOARN is to rapidly identify, recognize and respond to major international public health emergencies, and the challenges facing the international healthcare field remain daunting and require global cooperation and innovation (3). In 2024, the National Health Commission will fully promote the construction of close-knit county medical communities (4), further integrating medical resources in counties and realizing that the level of primary medical services is on a par with that of urban medical services. In recent years, there have been differences in the allocation of medical resources between different countries and between urban and rural areas (5). Problems such as Insufficient sinking of quality medical resources and lack of service capacity of primary hospitals, for example, the lack of access to emerging technologies in many community hospitals (6), which are still very serious in developing countries. Although medical institutions at all levels have vigorously invested in information technology, precise diagnosis and treatment services and the implementation of personalized health management are still facing certain difficulties. Traditional standardized treatment often ignores individual differences in patients’ genes, environment and behavior, and its effectiveness will be limited to a certain extent. In terms of health management, the health monitoring data provided by intelligent equipment can be used for the prevention or treatment of diseases, greatly saving medical costs, and more effectively responding to the severe challenges brought by the aging population (7). Human-computer collaboration technology can not only utilize the advantages of artificial intelligence in terms of speed and accuracy, but also effectively exert human intelligence, which is undoubtedly a wise move to deal with the existing medical dilemma. Human-computer collaboration refers to the intelligent perception, recognition and management of the information transfer process between things and people through the network, and is now widely used in intelligent manufacturing (8), intelligent transportation (9), intelligent life (10), intelligent medical (11) and many other aspects, and has achieved remarkable results. The purpose of this paper is to explain the concept of human-computer collaboration and the dilemmas faced by the global healthcare field through the search of previous literature, to explore the prospects of human-computer collaboration in the healthcare field and the challenges that may be faced during the development process, so as to alleviate the healthcare pressure in tertiary hospitals, to improve the imbalance in the structure of the global healthcare resource allocation, and to provide a new vision and insights for the development of the healthcare field’s intellectualization.

## 2 Methodology

### 2.1 Retrieval strategy

Being closely related to the research topic, these titles can accurately distill the core content of the article. Meanwhile, the comprehensiveness, security and authority of the selected databases are outstanding. They contain a variety of literature resources including national standards, industry standards, and Chinese and foreign standard catalog digest data, which provide solid data support for the study. Therefore, this paper is based on “Human-machine collaboration,” “Human-Computer Interaction,” “HCI,” “chronic disease,” “health management” and “precision medicine” subject terms, searched Chinese and English databases such as CNKI, Wanfang Data, VIP, CBM, PubMed, Web of science, Embase, Cochrane Library, etc., and the search time limit was from the establishment of the library to September 2024, and the English search formula was based on PubMed as an example, and the specific search formula were recorded in detail in Supplementary Table S1. For other databases, revisions were made using database-specific subject terms and keywords in the title and summary. The core idea of this search strategy is to focus on the two main themes closely related to this paper – health management and precision medicine, covering human-machine collaboration and its different titles, and including a series of common diseases defined as chronic diseases.

### 2.2 Literature inclusion and exclusion criteria

#### 2.2.1 Inclusion criteria

(1) research on human-computer collaboration in disease screening and diagnosis; (2) research on human-computer collaboration in health management; (3) research on human-computer collaboration in medical education; (4) research on human-computer collaboration in traditional medicine; (5) research on human-computer collaboration in medical data integration and preprocessing.

#### 2.2.2 Exclusion criteria

(1) duplicated and unrelated to the topic; (2) did not cover the application of human-computer collaboration technology in the medical field; (3) reviews and conference papers; (4) full text is not available; (5) the language is not Chinese or English.

For the exclusion criteria, redundancy in analysis and interpretation could be avoided and research efficiency ensured by excluding duplicative and topic-irrelevant literature; the exclusion of studies that did not involve the application of human-computer collaboration technology in medicine ensured that the focus of the study was on the specific application of human-computer collaboration technology in the medical field and excluded studies that may have involved human-computer collaboration technology but had no relevance to medicine; and the exclusion of review articles and conference papers, the as they may contain preliminary research or non-peer-reviewed work, which ensures a high level of scientific rigor in the included studies; the lack of access to the full text of the articles may result in an inability to fully assess the quality of the studies and to obtain all the necessary analytical data; exclusion of articles in languages other than Chinese or English as Chinese and English are two widely used languages covering a wide range of academic and professional literature. Restricting it to these two languages could expand the global applicability of the findings.

### 2.3 Literature screening

**Figure 1:**
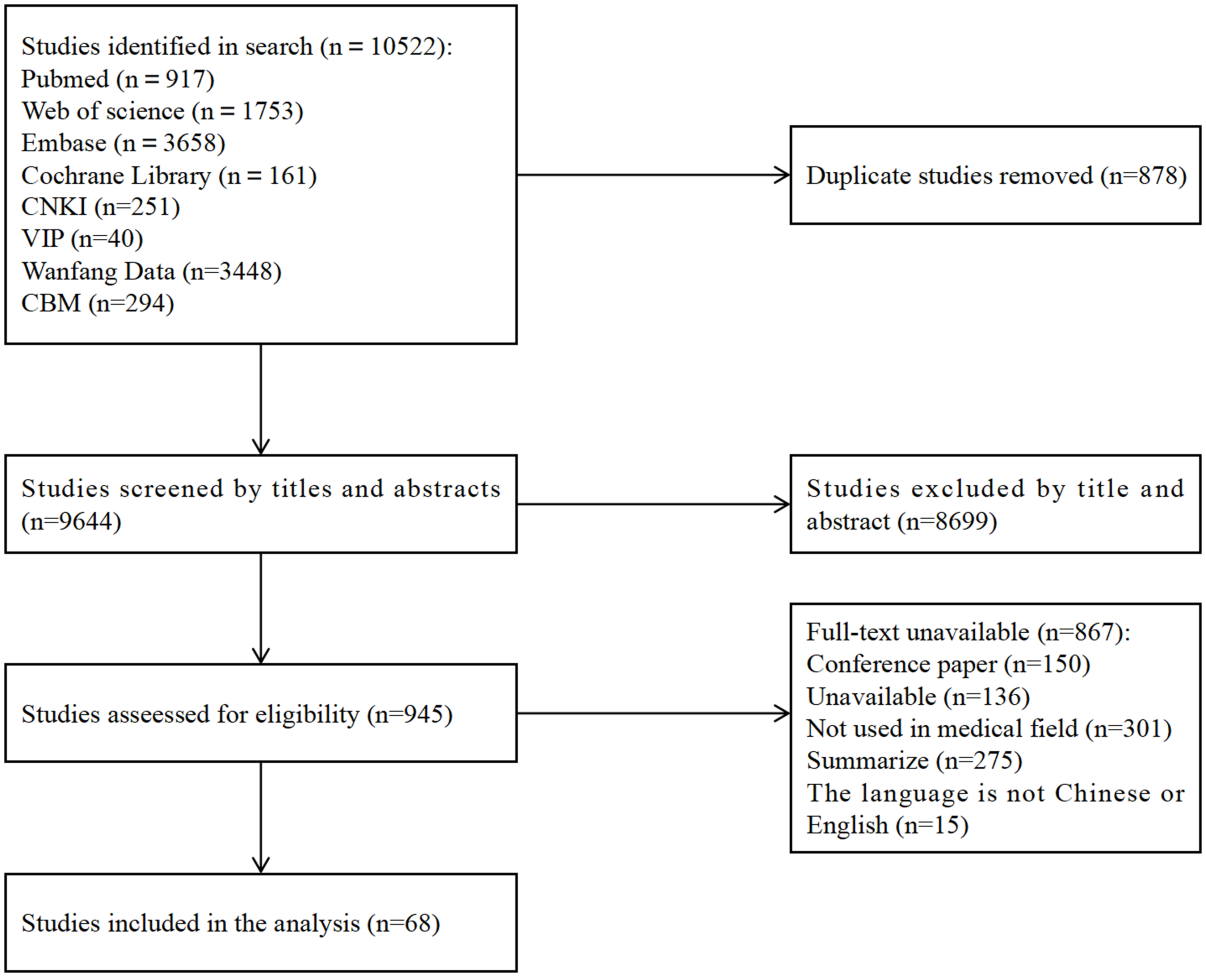
Flowchart of literature screening.

(1) Standardized process: A pre-set, standardized data extraction process will be used to reduce subjectivity. (2) Dual validation: Data extraction will be performed by two independent researchers to ensure the accuracy and consistency of the information. (3) Training and guidance: All personnel involved in data extraction will receive uniform training and follow a detailed instruction manual to ensure a common understanding of data extraction standards and procedures. (4) Resolution of differences: Differences in data extraction will be resolved through discussion. The search yielded 10,522 documents, and 68 relevant documents were finally included according to the inclusion and exclusion criteria, after an in-depth analysis of 68 relevant articles, we identified broad applications of HCI in five main areas: disease screening and treatment, health management, medical education, traditional medicine, and the integration and processing of medical data. As for other potential application areas, because it has not been fully developed and applied, it will not be further elaborated here. The literature screening process and results are shown in Figure 1.

### 2.4 Data extraction and quality assessment

Two independent evaluators (WW and LL) conducted title, abstract, and full-text evaluations using predetermined selection criteria to ensure methodological consistency with an inter-rater reliability of 80%. Extracted data included publication author, year, title, journal name, study design, setting, methods, and a summary of results and limitations. All disagreements were resolved through mutual discussion. All studies used the Mixed Methods Assessment Tool (MMAT) to consider risk of bias as it is applicable to all data types (12).

### 2.5 Data synthesis

In consolidating the findings, we first carefully assessed the methodological rigor of each study and the transparency of reporting to ensure the credibility of the results used. Second, we excluded studies of lower quality or with obvious biases to further validate the robustness of the results. In addition, we analyzed the design, sample size, year of implementation, and other key characteristics of the different studies to account for differences between the results. In the event of inconsistent results that are difficult to interpret, we invite experts in the field to provide specialized advice. We are committed to presenting all findings in the report in a transparent manner, including those with conflicting and inconclusive data, and to exploring possible reasons for these discrepancies. We also point out and discuss research limitations that may affect the interpretation of results. Finally, based on the conflicts and uncertainties in existing research, we suggest directions and recommendations for future research to promote further development of the field.

## 3 Overview of human-computer collaboration

In the process of advancing global integration and modernization in the era of digital intelligence, the world is experiencing profound changes led by the fourth scientific and technological revolution, and the continuous optimization and development of artificial intelligence is driving the continued progress of the healthcare industry. Human-computer collaboration, as an important extension of the field of artificial intelligence, is increasingly becoming an important force in promoting reform in the field of healthcare. Human-computer interaction can be simply understood as the process of communication and interaction between humans and machines or complex systems. When the “machine” in human-computer interaction is an intelligent “robot,” the system that interacts with people is an intelligent system, at which time the machine is able to understand and mimic human thinking and behavior, thus realizing more intelligent cooperation and decision-making, which is the connotation of human-computer collaboration (13).

## 4 Research status of human-machine collaboration

Human-Computer Collaboration (HCC) is becoming an increasingly mature and multidimensionally expanding field, and HCC technology is now widely used in surgical robots (14), diagnostic aids (15), health care (16), informationization (17), and other medical applications. However, there are still some gaps between developed countries and developing countries regarding the maturity and application breadth of human-computer collaboration technology development. The research on human-computer collaboration technology in developed countries started earlier, and its clinical application can be traced back to the end of the 20th century to the beginning of the 21st century (18), covering all levels from basic theoretical research to the development and application of medical equipment. The research on human-robot collaboration is also deepening, and the research field has expanded from traditional human-computer interaction (HCI) to human-computer intelligence collaboration, exploring how to realize effective collaboration between humans and intelligent systems. A variety of human-robot collaborative robots have been developed and applied in medical practices such as surgical operations, trauma treatment, and rehabilitation training (19), and significant progress has been made in digital healthcare technology. For example, the da Vinci robotic surgical system was first developed and put into use by an American company called Intuitive Surgical in 2000 (20), and was approved for introduction into China in 2006. The introduction of the da Vinci surgical robot has had a far-reaching impact on China’s healthcare sector, not only improving the quality of healthcare services, but also promoting the advancement of healthcare technology and the development of the healthcare industry. In 2022, Epione, a new interventional oncology robot for minimally invasive hepatocellular carcinoma treatment (21), was approved and put into use in the U.S. The birth of Epione interventional oncology robot marks a major advancement in interventional oncology technology, which, through human-robot synergy, provides doctors with a safer, more precise, and more efficient surgical tool, and is expected to improve the outcome and quality of life of patients. Secondly, developed countries are relatively leading in the standardization of human-robot collaboration technology, and have formed a set of more complete medical standard system, the American National Standards Institute released ANSI/RIA R15.06–2012 standard number (22) in 2012 as the national standard for industrial robots and robotic system safety requirements in the United States. In comparison, the completeness and rigor of the relevant standards in developing countries still need to be improved, and the international convergence and promotion of application still need to be strengthened. Not only that, economically developed regions have more in-depth research and regulation of medical ethics and legal issues involved in human-robot collaborative technologies, such as the White House released the Final Principles of Privacy and Trust in Precision Medicine and the Framework of Principles for the Security of Precision Medicine Programs in 2015 and 2016 (23), which are designed to ensure the confidentiality and integrity of precision medicine data while respecting participants’ preferences. The Korean government published a roadmap for regulatory reform (24) and decided to improve the regulatory system by uncovering 22 robotics-related regulatory issues in four application areas, including healthcare. Documents such as the World Medical Association’s (WMA) newly revised International Code of Medical Ethics (25) in 2022 provide ethical and other guidance for the global medical industry. In contrast, China’s research and regulation in these areas is still in its infancy, and in 2016 China’s National Health and Wellness Commission issued the Measures for Ethical Review of Biomedical Research Involving Human Beings (26), which stipulates in some detail the principles of medical ethical review, the focus of the review, and so on. Many governments have actively promoted the application of human-computer collaborative technology in medical technology through policy support and financial investment, and have promoted the research and application of related medical technologies, For example, the Japanese government is promoting the development of human-computer collaborative technologies through the “Society 5.0” strategy (27) and the Strategic Innovation Promotion Program (SIP) (28), especially in the medical and nursing care fields, in order to meet the challenges of an aging population. The South Korean government released the National Strategy for Artificial Intelligence, which includes applications in the medical field and aims to build South Korea into an AI powerhouse, with plans to create 455 trillion won in economic benefits by 2030. The Chinese government has introduced the New Generation Artificial Intelligence Development Plan (29), which aims to promote the innovation and application of AI technology and the development of human-machine collaboration. Policy support in developing countries is also increasing, but there is still room for improvement in the optimization of investment and policy environment. The research and application of human-machine collaboration technology is the focus of global attention. In general, although human-computer collaboration technology is developing in the direction of intelligence, naturalization and humanization from an international point of view, and is actively responding to the ethical and legal challenges brought about by technological progress, at the same time, after mastering the high technology of human-computer collaboration, developed countries, in order to consolidate their leading position in the global technological landscape, tend to adopt measures such as technological monopoly and restriction of technological output. This kind of behavior will bring about a series of problems, especially as there is a big gap between developing countries in terms of key technologies and knowledge reserves, resulting in a lack of necessary expertise and technical support. As a result, lagging countries may face problems such as insufficient technology output and uneven market distribution, which will not only limit their technological progress but may also exacerbate the widening of the global technology gap. In addition, the lack of policy and strategic planning for cooperative human-machine research may also hinder the systematic and long-term development of these countries in this field. Ultimately, this will not only deepen the technological divide between developed and developing countries, but may also trigger economic and social inequalities on a global scale. In this regard, we should not only pursue breakthroughs in the field of cutting-edge technology, but also realize common exploration, common development and common progress on a global scale. The Belt and Road Initiative proposed by China is committed to promoting common development among countries along the route and building the dream of global prosperity. This grand vision has always been an ideal that we cherish (30). At present, different countries and regions are also actively exploring its application in various industries through their own efforts, especially in the medical field, with the continuous progress of technology, it can be foreseen that human-computer collaboration technology will play a more important role in the medical field in the future.

## 5 Problems and current situation in the medical field

### 5.1 Inadequate and uneven distribution of high-quality medical resources

Globally, there is a serious imbalance in the distribution of health care resources. There are huge disparities in access to healthcare resources between developed and developing countries, between urban and rural areas, and between different socio-economic groups (31). African countries have strained healthcare resources, with less than 1 (32) doctor per 1,000 people, far below the 4 to 5 in developed countries, a serious medical brain drain, and a shortage of medicines (33). In Europe, universal healthcare services are an important part of the social welfare system in many countries. Take the National Health Service (NHS) of the United Kingdom as an example (34), which is a healthcare system covering the whole population and is committed to providing equal and high-quality healthcare services for all residents. The current problem is prevalent in many developing countries. The supply of medical and health resources in China has been increasing, and the capacity and efficiency of medical and health services have been improving, but the problem of underdevelopment of high-quality medical and health resources has not yet been fully resolved, especially at the grass-roots level and in the central and western regions (35). Insufficient professional talents in primary hospitals, insufficient medical service capacity, imperfect hierarchical diagnosis and treatment system, medical quality and safety issues, and insufficient investment in public health services (e.g., low penetration of cancer screening services in rural areas, and high demand for precision medicine and personalized health management) (36) are the main reasons why “it is difficult to see a doctor (37)” and “it is expensive to see a doctor.” The unequal distribution of health care resources will further exacerbate the imbalance in development between regions.

### 5.2 Inadequate medical management and protection system

The cost of health care in many countries continues to grow faster than gross domestic product (GDP), putting enormous pressure on health care systems. Despite the state-of-the-art technology in health care in the United States, the cost to patients is still quite high (38), which includes both direct healthcare costs and indirect insurance costs, and the high cost of healthcare has become a major problem facing the American society. As well as the commercial insurance-dominated healthcare system in the United States leads to inequality of services (39), and disadvantaged groups have difficulties in obtaining necessary medical care. From Asia’s perspective, China has made remarkable progress in its healthcare management and protection system, but there are still some problems, such as homogenization of the management of healthcare consortiums, health insurance coverage and reimbursement problems, the mechanism of guaranteeing the supply of medicines as well as the stability of prices have not yet been improved, as well as the guarantee of the construction of information technology in hospitals (40) and the digital transformation of primary healthcare is not sufficient (e.g., unreasonable increase in the cost of medicines, The level of standardization and normalization of medical services is not uniform, and failure to improve laws and regulations related to medical insurance), these problems will further exacerbate the contradiction between supply and demand of medical services.

### 5.3 Digital transformation in healthcare stymied

In the context of globalization, the limited health literacy of the public is the primary challenge facing the promotion of digital healthcare (41). Secondly, data barriers in different healthcare organizations may lead to duplication of patient visits, increasing the financial burden of patients and resulting in a waste of healthcare resources (42); the lack of a reasonable cost-sharing mechanism for digital healthcare services, especially in the application of enhancing the efficiency and level of established services, and the lack of clarity in the distribution of benefits have impacted the establishment of a cost-sharing mechanism (43). In addition, the lack of regulation of digital healthcare, including insufficient supervision, unclear service standards, and imperfect complaint and punishment mechanisms, has led to the phenomenon of “barbaric growth” in the development of the industry (44). In addition, there is a need to deepen the understanding of digital healthcare, including the role of technology providers, the understanding of the operating conditions of healthcare organizations, and the adaptability of service recipients, etc. These challenges will make it difficult to promote the in-depth application of digital healthcare technology in the healthcare field.

## 6 Application of human-machine collaboration in medical field

### 6.1 Application of human-machine collaboration in disease screening, diagnosis, and treatment

The application of artificial intelligence in disease screening and diagnosis has become one of the global research hotspots, which can accurately predict the occurrence, development and regression of diseases by imitating human cognitive functions, learning and analyzing massive amounts of structured and unstructured medical data (45), and fully mining and analyzing the features and laws of medical data (46). Human-machine collaboration has evolved and aided the diagnosis and treatment of disease. Zheng et al. (47) confirmed the advantages of human-computer collaborative technology in predicting the status of Epstein–Barr Virus (EBV) in gastric cancer slices, EBVNet’s average recipient area under the working curve (AUROC) from internal cross-validation was 0.969, AUROC was 0.941 on external datasets from multiple institutes, and AUROC was 0.895 on Cancer Genome Atlas datasets, which helps to effectively screen gastric cancer patients for immunotherapy with high economic benefits. Wang et al. (48) designed and promoted a human-computer interaction application and screening model for risk assessment of suspected symptoms online in response to the serious social and public safety problems caused by the novel coronavirus, which proved to be effective in alleviating social panic to a certain extent, avoiding the overly centralized use of medical resources, and decreasing the likelihood of cross-infection among patients. Krithika Rangarajan et al. (49) helped radiologists to differentiate between Coronaviral Virus Disease (COVID)-positive and COVID-negative patients using Chest X-ray (CXR) by using Convolutional Neural Networks (CNNs), a collaborative human-computer intelligence technique, and the results showed that the radiologists accuracy increased from 65.9 to 81.9% and the recall rate increased from 17.5 to 71.75. AI was effective in identifying AI demonstrated 92% accuracy in recognizing “normal” chest X-ray images as COVID-19. Min et al. (50) applied human-computer interaction technology to the diagnosis of children with autism spectrum disorders, and successfully improved the diagnostic efficiency with an accuracy rate of 80.5 to 95.5%, effectively overcoming the limitations of traditional diagnostic methods and verifying the significant effect of this technology in the diagnosis of autism. This study utilizes the advantages of human-computer interaction technology to provide new possibilities for early diagnosis and intervention through interaction with autistic children. The arm-type spine surgical assistance robot developed by the Shenzhen Institute of Advanced Technology of the Chinese Academy of Sciences (51) utilizes human–machine cooperative technology, multiple sensing technology, and the cooperation of multiple functional modules with high precision and high stability, and is capable of completing personalized and fine-perception surgical operations, achieving surgical safety control, and lowering surgical risks while giving the patient the maximum benefit of surgical recovery space. In order to cope with the many challenges of human-robot cooperative control in rehabilitation scenarios, a variety of flexible jointed exoskeleton robots are also gaining attention. Li (52) studied the system design of a hand-assisted exoskeleton robot to assist patients with hand dysfunction, such as stroke, spinal cord injury, and limb injury, to complete the manipulation of daily life objects, and centered on the human-computer interaction control, and ultimately verified the personalization of the assistive strategy and the validity of the active control method through experiments, which further confirms the practicability and efficiency of the method. The human-robot collaborative technology brings efficient and convenient screening and diagnosis for patients, which can maximize the utilization of medical resources and make up for the shortcomings of traditional treatment.

### 6.2 Application of human-machine collaboration in health management

The Population Culture Development Center of the National Health Commission of China launched the National Health Promotion Program (53), which emphasizes the importance of acquiring knowledge about healthy living and strengthening health education. For most of us, the resources needed for health management are also within reach. Health management (54) is a multifaceted and comprehensive process that helps to prevent disease, improve quality of life, reduce healthcare costs, prolong healthy lifespan, and promote the overall well-being of individuals and society. The application of man-machine collaboration technology in the field of health management has been supported by mature technology. Human-computer collaboration can be used for real-time monitoring, predictive analytics, and early warning systems in healthcare environments, based on algorithms that predict patient deterioration, adverse events, and readmissions, leading to proactive interventions and personalized health management. Monteiro-Guerra Fet al (55) developed an innovative app for survivors of breast cancer treatment. The app will provide personalized feedback, guidance, and motivation to encourage patients to be physically active, thereby promoting a healthy lifestyle. Through a well-designed interactive interface and intelligent algorithms, the app is able to provide customized exercise plans and health advice based on each user’s unique needs and recovery progress. The results of the study proved that the app can significantly improve the quality of life of breast cancer survivors. Population aging has become a pressing reality for today’s society, with far-reaching impacts on the economy, society, and healthcare system, and the Chinese government has made clear its strategy for dealing with it in the Outline of the National Medium-and Long-Term Plan for Actively Responding to Population Aging (56), in order to improve the basic older adult care service system. Human-computer collaborative technologies have brought new perspectives and insights to the field of health management for the older adult. Zhen ZZ et al. (57) designed a human-computer interactive noninvasive cardiovascular health assessment system that can dynamically monitor key physiological parameters such as blood pressure, blood flow, and blood glucose in older adult patients, which provides an innovative approach to assessing and managing cardiovascular health conditions. By monitoring and analyzing these parameters in real time, the system has the ability to identify potential threats to cardiovascular disease at an early stage and can proactively alert healthcare professionals to ensure that they can respond and take action quickly to effectively reduce the risk of acute morbidity. In order to improve the effectiveness of human-computer interaction medical devices in monitoring the human body in sedentary and bedridden states, Daiwili (58) constructed a human posture recognition system based on wireless body area network technology, and designed an optimized artificial neural network to integrate with it, and then applied this innovative system to human-computer interaction medical devices. The results show that the recognition accuracies of the human-computer interaction medical devices under this method are all significantly higher than 90%, with high recognition accuracy and efficiency, which provides a more solid technical support for the application of human posture recognition and related medical devices. Niu et al. (59) proposed a multimodal fusion health monitoring method based on a deep learning model to comprehensively and accurately assess the health status of individual patients, thereby improving diagnosis, intervention, and overall healthcare outcomes, and experimentally confirmed that the multimodal fusion method based on a deep learning model outperforms the existing health detection methods in terms of capturing complex patterns and predicting the health status, which validates the study. The validation of this study marks the beginning of a new era in the field of tele-health monitoring, where the accuracy and efficiency of human gesture recognition and related medical devices will be improved. The validation of this study marks a revolutionary advancement in the field of remote health monitoring, and is a harbinger of a future in which human-computer intelligent interactions will be used to provide more accurate and personalized healthcare services. The companion robot (60) developed by the Institute of Advanced Industrial Science and Technology in Japan analyzes the patient’s physiological data in real time (61, 62), records the patient’s emotions, interacts and communicates with the patient, and provides emotional support and psychological counseling in a timely manner, in order to alleviate the mental stress and loneliness due to the disease, as well as to improve the patient’s quality of life and enhance the efficacy of the treatment. The study of Andreallo (63) also confirms that patients who are lacking in interpersonal companionship patients can indeed feel attention, reliability, support, trust, and goodwill from the companion robot, resulting in a sense of real companionship. The application of human-computer intelligence and collaboration technologies in health management not only improves the quality and efficiency of healthcare services, but also shows great potential in personalized medicine and resource optimization, and provides new insights for coping with an aging society. It is summarized in Table 1.

**Table 1:** Health management case summaries.

Author/country	Project	Contents	Participates	Significance
Monteiro-Guerra et al. (55), USA	Application program	Personalized feedback, guidance, and incentives encourage patients to actively participate in physical activity	Survivors of breast cancer treatment	Significantly improve the quality of life for breast cancer survivors
Zhen (56), China	Non-invasive cardiovascular health assessment system	The key physiological parameters such as blood pressure, blood flow and blood glucose were dynamically monitored in older adult patients	Gerontal patient	Early identification of potential cardiovascular disease threats
Dai (57), China	Medical monitoring equipment	Human posture recognition and related indicators	Sedentary, bedridden patients	The accuracy was significantly higher than 90%
Niu (58), China	Multi-modal fusion health monitoring method based on deep learning model	A comprehensive and accurate assessment of the patient’s health status	Patient	It is superior to the existing health detection methods
Industrial Technology Research Institute (59), Japan	Escort robot	Provide emotional support and psychological counseling to improve patients’ quality of life and improve outcomes	Lonely patient	Relieve mental stress and loneliness caused by illness

### 6.3 Application of human-machine collaboration in medical education

“China’s Education Modernization 2035” (64), promulgated by the Political Bureau of the Central Committee of the Party of China, General Office of the State Council in February 2019, emphasizes universal education and accelerating the reform of education in the information age, highlighting the importance of information education. Human-robot collaborative teaching broadly refers to the coordinated integration of teaching elements in the “two-teacher classroom”, and narrowly refers to the collaboration between teachers and AI educational robots (65). However, the application of artificial intelligence in education has a history of more than thirty years (66), with the evolution of the times, human-computer collaborative intelligent education should be born. Zhang et al. (67) designed and verified the accuracy of the Virtual Standardized Patient simulator and addressed the limitations of Standardized Patient (SP) in history taking through the Virtual Standardized Patient (VSP), where students complete an exam involving the obtaining an SP history while the VSP provides real-time scoring. The results show that using voice and text recording as a guide and confirming that the VSP has a comprehensive and detailed scoring system and exhibits good scoring accuracy, it can be an important aid for students in developing history taking and history taking skills and greatly enrich their learning experience. Pang et al. (68) designed an immersive, real-virtual intelligent learning space where learners can choose their own identity after entering the learning space and other roles are played by other learners or robots. They need to assess, take measures, check and reassess the patient’s condition and the human-robot intelligent collaborative system provides the learners with recommendations for condition assessment in the process, assists in the formation of decisions and guides the subsequent treatment, and in the whole working process, the system is not only applied to the clinical practice, which serves as a common decision-making and collaborative operation, but also records the learner’s operation through multimodality. The standardized operational demonstration of the human-computer intelligence collaborative system motivates and standardizes the learners, significantly enhancing their motivation and skills (69). den Harder et al. (70) introduced e-learning (71) in undergraduate radiology education and improved students’ knowledge and skills in radiology through the use of human-computer interaction, and the experimental results confirmed that the use of this method can significantly improve the learners’ knowledge of radiology and image interpretation skills. Zhang et al. (72) introduced a stroke computer simulation system in neurology clinical teaching, using virtual reality technology to simulate the clinical symptoms, diagnosis and treatment process of stroke patients, allowing students to learn pathophysiological knowledge in a simulated environment through human-computer interaction, and visualizing theoretical knowledge. The study shows that this simulation training helps students understand the clinical features of stroke, improve their ability to analyze and solve clinical problems, and understand the individualized needs of clinical treatment. As the application of human-computer collaboration technology in the field of education continues to deepen, it not only promotes the integration and development of educational technology, but also promotes the innovation of the teaching model, realizes personalized learning, and provides a richer and more realistic learning experience through virtual reality and artificial intelligence technology. In particular, the new technological revolution under the arrival of the artificial intelligence era heralds a change in the paradigm of medical education. The shift from “disease-centered” to “health-centered” requires medical education to update its concepts, innovate the talent training system, and establish a new paradigm of education oriented to health needs.

### 6.4 Application of human-machine collaboration in traditional Chinese medicine

Currently, China is committed to accelerating the application and development of Chinese medicine in the medical field. The Outline of the Strategic Plan for the Development of Traditional Chinese Medicine (2016–2030) (73) promulgated by the Political Bureau of the Central Committee of the Party of China, General Office of the State Council in June 2016, emphasis is placed on adhering to the equal importance of Chinese and Western medicine, implementing the equal status of Chinese medicine and Western medicine, following the law of development of Chinese medicine, expanding the service areas of Chinese medicine, promoting the combination of Chinese and Western medicine, and promoting the revitalization and development of the cause of Chinese medicine in a coordinated manner. The application of human-computer collaborative technology in the field of traditional medicine is promising and has great potential for development. Wang et al. (74) designed an interactive data mining framework based on TCM identification of pediatric pneumonia using a three-layer model, which promotes the combination of data mining technology and modernization of traditional Chinese medicine through human-computer interaction while mining information. The establishment of this framework not only promotes the standardized development of TCM identification methods, but also builds a solid platform for in-depth analysis and modernization exploration of TCM information. Xie (75) developed a training and assessment system for Chinese medicine identification through the human-computer interaction process of building applications, simulating clinical practice scenarios by reproducing real clinical cases, thus helping teachers and students to consolidate their book knowledge and strengthen their clinical skills training, and the practical application shows that the intelligent platform based on human-computer interaction technology has a lot of room for application and promotion of students’ identification ability training. The pulsation formed by the interaction between the heart contraction and diastole of the human cardiovascular system and the various resistances encountered during the flow of blood along the blood vessels is named “pulse wave,” which reflects the characterization and mapping of the internal movement state of the human heart. TCM pulse diagnosis, which diagnoses the health condition of the human body by observing the pulse waveform, has a long history and an extremely important position in TCM. With the development of modern science and technology, the application of human-computer collaborative technology is expected to further promote the modernization and standardization of TCM pulse diagnosis, and provide a more scientific and objective means of analysis for TCM pulse diagnosis. Wang (76) realized the effect of human-computer interaction by designing two parts, the terminal pulse acquisition system and pulse classification, and loading the integrated display, which can display the pulse waveform and pulse results in real time. The experimental results show that the wearable pulse collection system developed in this research is closely integrated with TCM pulse diagnosis with an accuracy rate of 91.67%, and this achievement not only meets the needs of TCM for the diagnosis of subhealth state, but also provides a strong support for the modernization of pulse diagnosis and the inheritance of TCM academics. Zhang et al. (77) designed a user-friendly TCM tongue analyzer equipped with an intuitive and efficient interactive interface, aiming to provide users with a convenient and comfortable operating experience. Clinical applications have demonstrated that the user-friendly human-computer interface plays a key guiding role in facilitating the objectification, quantification and standardization of TCM tongue diagnosis. Human-computer collaboration technology also faces many challenges in the process of its wide application in the field of Chinese medicine. Firstly, it is a technical problem to combine the complex TCM and its individualized treatment plan with modern computer technology. Secondly, the ambiguity of TCM terminology, the obscurity of theoretical knowledge, the abstraction of therapeutic thinking, and the complexity of TCM medical records have brought great obstacles to the modernization of TCM. In addition, TCM diagnosis involves a complex process of evidence typing, which requires the integration of multiple data sources. However, the current publicly available TCM-related datasets are not only limited in quantity, but also of varying quality, which severely restricts the training and validation of intelligent evidence-based typing models. These are still the direction of our efforts. Human-computer collaborative technology brings new opportunities and challenges to the development of TCM, and through continuous exploration and innovation, the unique advantages of TCM can be fully utilized to make greater contributions to the cause of human health.

### 6.5 The application of human-machine collaboration in medical data integration and preprocessing

At present, the problem of “medical data silos” (78) has not yet been resolved in a breakthrough manner, and it is still difficult to realize the trustworthy circulation across hospitals of most health information systems of medical institutions, including patients’ electronic medical records and diagnostic imaging reports, etc. Therefore, there is an urgent need to promote the development of new smart healthcare service models by integrating large-scale multi-source heterogeneous multi-modal medical data resources across regions, communities and organizations (79). The human-computer collaborative case base is one of the important achievements (80). Under the guidance of physicians, the system forms high-quality case evaluation criteria by automatically learning from historical data to realize large-scale case quality assessment. At the same time, the number of cases in the case base increases with time and medical institutions, and the system rapidly accumulates knowledge and realizes the process of autonomous learning, which improves the system’s reasoning ability and the accuracy of knowledge discovery. The system realizes the functions of comprehensive medical and healthcare knowledge service in large general hospitals, community-assisted diagnosis and treatment, and clinical teaching and research assistance, helping the implementation of the national strategy of hierarchical diagnosis and treatment and healthy China. With the development of hospital informatization level, the traditional single machine learning method to analyze information data is not accurate enough for safe decision-making, which makes intelligent human-machine collaboration technology quietly emerging. Chen et al. (81) confirmed that human-computer interaction and collaboration is achieved through the human-computer collaboration algorithm distributed forwarding card, using computer artificial intelligence in information processing with the operator at the control end to coordinate and cooperate, so that it is highly accurate and responsive in the process of information storage, access, etc., and can accurately detect or supervise and feedback the dynamic perceptual information of the hospital to the human-computer interaction interface (82), assisting the operator to make accurate judgment and enhance the hospital information security level and system functionality. Image segmentation is a ubiquitous step in almost any medical image research, Ravanbakhsh M et al. (83) proposed a conditional generative adversarial network based approach to address the generator problem in generative adversarial networks by combining segmentation of unlabeled data with human-computer interaction modes of operation synthesis in a semi-supervised learning environment and using a discriminator to identify unreliable slices that require expert annotation. The results show that the method provides a level of evaluation at the slice level that is comparable to top fully supervised techniques. Ding et al. (84) proposed a design method for hospital health checkup information retrieval system based on Unified Modeling Language (UML) and embedded multithreading technology, constructed the information sampling module and human-computer interaction platform of the information management system, realized the preprocessing of hospital health checkup information retrieval, and finally carried out simulation analysis. The results show that intelligent retrieval of hospital health checkup information using this method can not only significantly improve the accuracy rate, but also significantly improve the retrieval efficiency. The application of this technology can not only build the knowledge graph and inference engine of clinical big data, and deeply excavate the intrinsic connection between disease symptoms, but also realize the data exchange and information sharing between medical institutions, and between institutions and patients, so as to build a synergistic development of the medical service ecosphere. This not only promotes the digital and intelligent transformation of medical services, but also injects new momentum into the future development of the entire medical field.

## 7 Challenges and prospects

With the continuous development of artificial intelligence technology and the continuous intermingling of cross-disciplinary technologies, human-computer collaboration technology, although widely used in the medical field, still faces many bottlenecks that are difficult to break through. Especially in the deep learning stage of artificial intelligence, for some diseases, it is difficult to obtain enough data, which limits its application in disease screening and makes the division of responsibility for medical accidents complicated. We need to clarify the role and responsibility of AI in medical decision-making. In addition, how to combine the individualized treatment plan of TCM with modern computer technology is also a technical challenge. Data integration is difficult due to the lack of unified metadata standards. Meanwhile, due to the lack of unified management of master data, it is impossible to uniquely identify and update the core data entities of a patient or a doctor during the medical process. A large amount of unstructured data exists in the form of text, images, images, etc., which increases the difficulty of data integration and analysis. The development and design of AI technologies may be influenced by the designer’s own values, which in medical education may lead to latent value discrimination and bias the data collected by algorithms for teaching needs. All of these challenges need to be thoroughly researched and addressed to ensure the healthy development of human-computer collaborative technologies in the medical field. Secondly, human-machine collaboration technology is currently in the initial development stage, and technical constraints and development costs (85) are an important aspect, and existing human-machine collaboration technologies still have limitations in handling complex tasks and adapting to uncertain environments. However, due to the lack of appropriate professional knowledge and intuitive judgment of the machine, it may show bias according to the characteristics of the patient when performing intelligent analysis, and if the algorithms have a preference for a specific individual or group of people (86). To a large extent, this may adversely affect the interests of other groups, while the complexity of the algorithms leads to less interpretability and transparency (87), Min et al. (88) discussed in their research that artificial intelligence (AI) systems do have multi-faceted problems such as algorithmic bias, which seriously affect the fair rights and interests of users and it is difficult for doctors and patients to understand these algorithms decision-making process. In addition, Lukyanenko R’s research shows that communication and trust is another challenge in human-machine collaboration. Effective communication between humans and machines needs to overcome the barriers of verbal communication and information sharing, and the level of trust in the new paradigm of collaborating with machines has yet to be improved (89). Many older adult patients have low levels of e-health literacy (90) and are cautious or even skeptical of emerging technologies, According to the results obtained by Jiang X et al. (91) through meta-integrated data, the eHealth literacy score of the older adult was 21.45 (95% CI: 19.81–23.08). Subgroup analysis showed that among older age groups, e-health literacy was lower in women 19.13 (95% CI: 15.83–22.42) and in people aged 80 years and older 16.55 (95% CI: 11.73–21.38), older adults without spouses and living alone had lower e-health literacy 18.88 (95% CI: 15.71–22.04) and 16.03 (95% CI: 16.51–21.79), a situation exacerbated by the complexity of the operating technology. Meanwhile, security and privacy issues are also aspects of human-computer collaboration technology that cannot be ignored (92). Human-computer collaboration usually requires the sharing of large amounts of data and information, which involves issues of data security and personal privacy protection (93). Poli et al. (94) conducted an in-depth discussion on the ethical issues of AI through literature review and case analysis, and reported the ethical challenges caused by the development and application of generative AI technology, especially prejudice, privacy invasion, unclear responsibility, and the spread of false content. However, human-computer collaboration technology plays an important role in improving the efficiency and quality of medical services, promoting the scientificization of medical decision-making, accelerating the development of medical research and education, reducing the workload of medical personnel, and enhancing the rehabilitation experience of patients. In addition, it also promotes the innovation of medical service model. Many governments around the world have issued policy documents to support the application of AI technology in clinical decision support, patient monitoring and guidance, and assisted surgery to promote the process of smart healthcare. Human-computer collaboration research also requires close cooperation of interdisciplinary teams, bringing together experts from different fields to participate in system design and ensuring a balance between technical aspects and humanistic care. In the medical field, human-computer collaboration is not only of great practical significance, but also of far-reaching strategic value, which not only opens up new possibilities for the development of smart healthcare, but also promotes the technological innovation and service model transformation and upgrading of the medical industry.

## 8 Evaluation of limitations and suggestions for future studies

### 8.1 Limitations

This paper is a narrative review, the quality of which depends heavily on the author’s ability to interpret and analyze existing research findings. This dependence may have a subjective impact on the conclusions and overall narrative of the review, thus compromising its objectivity and accuracy. If the review fails to provide an adequate overview and in-depth analysis of historical research findings, and instead simply summarizes past views, it may lack a critical examination of research flaws. In addition, the lack of clear breakthroughs and innovations is also a common problem with narrative reviews, which limits their potential to promote progress and innovation in the academic field. The search strategy of this paper is mainly based on Chinese and English sources and is limited to some Chinese and English databases, which may lead to problems of language preference, database coverage, and uneven number of references. Relying on limited database resources may result in an incomplete collection of literature, thus affecting the generalizability and accuracy of the research results. However, despite our exhaustive screening process, potential biases caused by human factors are inevitable in the operation. Researchers may unconsciously tend to select information that supports their preconceived hypotheses, and this tendency may inadvertently introduce bias, which in turn affects the representativeness of the sample and the objectivity of the study. Despite the careful design of the inclusion and exclusion criteria, these exclusion criteria may have biased the results. Due to the exclusion of non-Chinese and English studies, key findings in the literature in other languages may have been omitted, thus limiting the generalizability and applicability of the findings. At the same time, excluding studies without full text may inadvertently introduce publication bias, especially for important research findings that may have been published in less influential journals. In addition, restricting the language of research may introduce cultural and geographical bias, as research findings from certain regions may be published mainly in literature other than Chinese or English, which may affect the comprehensiveness and balance of the findings.

### 8.2 Suggestions for future studies

In order to overcome and address bottlenecks in the development of HCM, we need to pay special attention to areas that are underrepresented in the integration of HCM. This includes in-depth research on the potential and effectiveness of human-computer systems in improving access to healthcare in rural and resource-poor areas. We advocate longitudinal studies and recommend long-term studies to more accurately assess the sustainability and scalability of these technologies in the real world. In addition, we recommend comparative studies that explore the effectiveness of these technologies in both developed and developing countries. At the same time, we call for more user-centered research aimed at improving the interface design of human-computer systems, increasing their accessibility and user acceptance, and directly addressing health literacy and lifelong health learning. Through these combined efforts, we can more effectively promote the development of human-computer collaboration technologies and ensure their fair and effective application on a global scale. Looking ahead, human-computer collaborative intelligent systems are developing in the direction of personalization, remoteness, and intelligence. These systems will play an important role in areas such as intelligent decision-making for clinical diseases, medical image analysis and quality control, radiotherapy planning and scientific research. In addition, they will innovate intelligent outpatient, medical consultation, pre-diagnosis, in-diagnosis and post-diagnosis services, realizing whole life cycle health management and providing patients with more accurate and efficient medical services. Not only that, in many countries, the application of human-computer collaboration technology also has many problems such as research and development costs, patient acceptance, information security, legal ethics, technical bottlenecks and so on, which leads to the limitation of the application of human-computer collaboration technology in the medical field. Therefore, the development and promotion of human-computer collaboration technology in the future should take into account the above limitations to ensure that the application of human-computer collaboration in the medical field can not only promote the development of medical technology, but also maximize the protection of the rights and interests of patients, so as to achieve the optimal allocation of healthcare resources and reform of the healthcare system.

## 9 Conclusion

Human-computer collaboration has revolutionized the healthcare field, especially in disease review, diagnosis, health management, and education. This collaboration has greatly optimized the process of patient treatment, prognosis and recovery, significantly enhanced the healthcare experience, and played a crucial role in driving innovation in healthcare technology. Looking ahead, human-computer collaboration is expected to further promote global health equality through continuous innovation and breakthroughs, and to spread advanced healthcare services to all parts of the world, especially in areas where healthcare resources are scarce and healthcare management systems are not yet well-developed. The continuous development of human-computer collaboration will surely provide a strong impetus for the common prosperity and progress of people around the world.
