# Supplementary material for: Advances in the application of human-machine collaboration in healthcare: insights from China
Source: Front Public Health. 2025 Feb 5;13:1507142. doi: 10.3389/fpubh.2025.1507142 (PMC11835885; doi:10.3389/fpubh.2025.1507142)
Supplement: Supplementary file 1 [file Table_1.DOCX]

Table S1. Electric search strategy in PubMed

| Step | Search Details | Results |
| --- | --- | --- |
| #1 | human-computer collaboration[Title/Abstract] OR human-computer interaction[Title/Abstract] OR "HCI"[Title/Abstract] | 4246 |
| #2 | ("Chronic Disease"[Mesh]) OR ("Hypertension"[Mesh]) OR ("Myocardial Ischemia"[Mesh]) OR ("Heart Failure"[Mesh]) OR ("CORonary Disease"[Mesh]) OR ("Asthma"[Mesh]) OR ("Pulmonary Disease, Chronic Obstructive"[Mesh]) OR ("Diabetes Mellitus"[Mesh]) OR ("Neoplasms"[Mesh]) OR ("Stroke"[Mesh]) OR ("Gastritis"[Mesh]) OR ("Peptic Ulcer"[Mesh]) OR ("Enteritis"[Mesh]) OR ("Nephritis"[Mesh]) OR ("Kidney Failure, Chronic"[Mesh]) OR ("Osteoarthritis"[Mesh]) OR ("Precision Medicine"[Mesh]) | 6,412,830 |
| #3 | chronic[Title/Abstract] OR "long term condition"[Title/Abstract] OR hypertension[Title/Abstract] OR hypertensive[Title/Abstract] OR "high blood pressure"[Title/Abstract] OR "elevated blood pressure"[Title/Abstract] OR cardiovascular[Title/Abstract] OR cardio-vascular[Title/Abstract] OR "CVD"[Title/Abstract] OR heart[Title/Abstract] OR cardiac[Title/Abstract] OR cORonary[Title/Abstract] OR cardiopulmonary[Title/Abstract] OR respiratORy[Title/Abstract] OR pulmonary[Title/Abstract] OR lung[Title/Abstract] OR asthma[Title/Abstract] OR "chronic obstructive pulmonary disease"[Title/Abstract] OR "COPD"[Title/Abstract] OR "chronic obstructive lung disease"[Title/Abstract] OR diabetes[Title/Abstract] OR diabetic[Title/Abstract] OR "DM"[Title/Abstract] OR hyperglycemia[Title/Abstract] OR hyperglycaemia[Title/Abstract] OR cancer[Title/Abstract] OR carcinoma[Title/Abstract] OR malignancy[Title/Abstract] OR tumOR[Title/Abstract] OR tumour[Title/Abstract] OR oncolog*[Title/Abstract] OR stroke[Title/Abstract] OR "cerebrovascular accident"[Title/Abstract] OR "CVA"[Title/Abstract] OR dementia[Title/Abstract] OR alzheimers[Title/Abstract] OR "cognitive impair*"[Title/Abstract] OR gastr*[Title/Abstract] OR stomach[Title/Abstract] OR intestin*[Title/Abstract] OR renal[Title/Abstract] OR kidney[Title/Abstract] OR osteo*[Title/Abstract] OR "individualized medicine"[Title/Abstract] OR "personalized medicine"[Title/Abstract] OR healthcare[Title/Abstract] OR "health management"[Title/Abstract] | 11,107,017 |
| #4 | #2 OR #3 | 12,716,213 |
| #5 | #1 AND #4 | 917 |
